# Mechanical Properties of the Carbon Nanotube Modified Epoxy–Carbon Fiber Unidirectional Prepreg Laminates

**DOI:** 10.3390/polym13050770

**Published:** 2021-03-02

**Authors:** Gökhan Bakis, Jan-Felix Wendel, Rico Zeiler, Alper Aksit, Markus Häublein, Martin Demleitner, Jan Benra, Stefan Forero, Walter Schütz, Volker Altstädt

**Affiliations:** 1 Department of Polymer Engineering, University of Bayreuth, Universitätsstraße 30, FAN A, 95448 Bayreuth, Germany; goekhan.bakis@basf.com (G.B.); jfwendel@gmx.de (J.-F.W.); rico.zeiler@zf.com (R.Z.); alper.aksit@uni-bayreuth.de (A.A.); markus.haeublein@uni-bayreuth.de (M.H.); martin.demleitner@uni-bayreuth.de (M.D.); 2 FutureCarbon GmbH, Ritter-von-Eitzenberger-Straße 24, 95448 Bayreuth, Germany; jan.benra@future-carbon.de (J.B.); stefan.forero@future-carbon.de (S.F.); walter.schuetz@future-carbon.de (W.S.)

**Keywords:** carbon nanotubes, epoxy resins, carbon fibers, nanocomposites, prepregs, fiber-reinforced composites, toughness, mechanical properties, aerospace

## Abstract

The effect of plasma treatment of the multi-walled carbon nanotube (MWCNT) surface on the fracture toughness of an aerospace grade epoxy resin and its unidirectional (UD) carbon fiber prepreg laminates has attracted scientific interest. A prepreg route eliminates the possible risk of carbon nanotube filtration by unidirectional carbon fibers. X-ray photoelectron spectroscopy results suggested that oxygen atom concentration at the nanotube surface was increased from 0.9% to 3.7% after plasma modification of the carbon nanotubes. A low number (up to 0.5 wt.%) of MWCNTs was added to epoxy resin and their carbon fiber prepreg laminates. Transmission electron micrographs revealed that the plasma treatment resulted in a better dispersion and distribution of MWCNTs in the epoxy resin. Plasma-treated MWCNTs resulted in a more pronounced resistance to the crack propagation of epoxy resin. During the production of the reference and nanotube-modified prepregs, a comparable prepreg quality was achieved. Neat nanotubes agglomerated strongly in the resin-rich regions of laminates lowering the interlaminar fracture toughness under mode I and mode II loading. However, plasma-treated nanotubes were found mostly as single particles in the resin-rich regions of laminates promoting higher energy dissipation during crack propagation via a CNT pull-out mechanism.

## 1. Introduction

Since the last decade, fiber-reinforced polymer composites have been increasingly used by the civil aircraft industry due to their high specific stiffness and strength, chemical resistance, and thermo-mechanical properties [1]. Carbon fiber prepregs impregnated with epoxy resins have especially been the material of choice for the primary structural composite parts. It is, however, well known that epoxy resins are generally brittle and possess low fracture toughness [2]. The toughening of epoxy resins has been widely investigated by incorporating various additives in matrix, such as core–shell [3], rubber [4], nano-silica [5], layered silicates [6,7], graphene [8], and single- or multi-walled carbon nanotubes [9,10,11].

Among those, carbon nanotubes (CNTs) are very promising nano-additives for mechanical enhancement of the host polymer due to the exceptional mechanical properties of the single carbon nanotube particles [12,13,14]. A high number of parameters, including the dispersion and distribution quality of CNTs [15], or the nanotube length and aspect ratio [16] affect the nanocomposite morphology, and therefore the final mechanical properties. The matrix–nanotube compatibility especially plays a crucial role regarding the toughening efficiency of these particles [17]. Gojny et al. [17] studied the effect of the surface functionalization of CNTs with primary amines on the toughness of an epoxy resin. Although the incorporation of CNTs in general led to an enhancement of the fracture toughness, functionalized CNTs showed an improved dispersion and a higher matrix compatibility, leading to a further enhanced toughness [17].

Carbon nanotubes have been incorporated to toughen the endless fiber-reinforced composites (FRCs) [18,19,20,21] or induce multifunctionality [22]. Bekyarova et al. [19] grew CNTs on a carbon fiber surface and processed the fibers with an epoxy resin via vacuum-assisted resin injection molding. It was observed that the CNT localization at the fiber–matrix interphase resulted in a 30% higher interlaminar shear strength compared to the reference laminate. Garcia et al. [20] introduced a neat CNT-forest as an interleaf with the hand lay-up processing to the interlaminar region of an aerospace grade prepreg laminate, which then led to a tremendous increase of toughness under mode I and mode II loading.

To best of our knowledge, it is very challenging to investigate only the effect of CNTs surface functionalization on the mechanical properties of nanotube-modified fiber composites, since the final composite properties are highly sensitive to the composite quality and the fiber volume content. In addition, a possible nanotube filtration by endless reinforcing fibers during resin transfer molding or infusion can hinder a systematic study of CNTs with different surface chemistry in the fiber-reinforced composites. Zeiler et al. [21] studied the effect of the diglycidyl ether of bisphenol-A (DGEBA) molecule as a surface modifier for multi-walled carbon nanotubes (MWCNTs) and investigated the mechanical properties of their glass fiber-reinforced epoxy composites. Only functionalized MWCNT-modified resin could be processed with non-crimp, stitch-bonded unidirectional glass fibers via resin transfer molding since neat CNTs increased the resin viscosity and showed the risk of filtration by glass fibers. 

Within the scope of this work, the main aim was to understand the effect of surface modification on the dispersion and distribution of multi-walled carbon nanotubes in unidirectional carbon fiber–epoxy prepreg laminates, which were then correlated to their interlaminar fracture toughness. X-ray photoelectron spectroscopy revealed the surface atomic concentration of nanotubes before and after plasma treatment. A linear elastic fracture mechanics approach was employed to study first the effect of MWCNTs on the fracture toughness of the epoxy resin. To investigate the effect of the nanotube dispersion and surface modification in prepreg laminates, it was crucial to prohibit any filtering of nanotubes by fibers during composite processing. Therefore, in this work, the extrusion process of nanotubes in resin and following hot melt prepreg route were employed to eliminate any filtering of nanotubes via fibers. These advanced dispersion and composite processing routes allowed a detailed investigation of the effect of surface modification of CNTs and the resulting dispersion of nanotubes in prepreg laminates without any constraint of fibers. Finally, the fracture toughness of nanocomposites and nanotube-modified prepreg laminates was investigated and correlated with the composite morphology. 

## 2. Materials and Methods

### 2.1. Materials 

**Figure 1:**
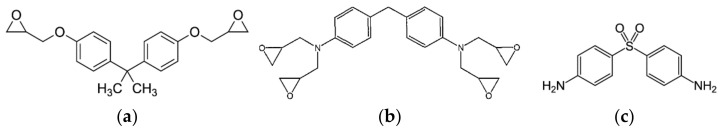
(**a**) DGEBA resin, (**b**) TGMDA resin, and (**c**) 4,4′-DDS hardener.

The epoxy blend formulated for this study consists of the diglycidyl ether of bisphenol A (DGEBA) resin, Baxxores 2200^®^ (epoxy equivalent weight: 182 g.mol^−1^, provided by BASF, Ludwigshafen, Germany) and tetraglycidyl methylene dianiline (TGMDA) resin, Epikote^TM^ 496 (epoxy equivalent weight: 115 g mol^−1^, which is provided by HEXION, Duisburg, Germany) in ratio of 40 and 60 phr of the resin part, respectively. As hardener, 4,4′-diaminodiphenyl sulfone (4,4′-DDS) ORGANICA^®^ (amine equivalent weight: 62.075 g.mol^−1^ provided by Feinchemie GmbH, Bitterfeld-Wolfen, Germany) was chosen and added stoichiometrically to the resin blend. Dry and hot–wet glass transition temperature (hot–wet: after 14 days immersed in 70 °C water) are 219 °C and 201 °C, respectively, which are measured via dynamical mechanical thermal analysis (DMTA, 3K/min, 1 Hz, 0.1% deformation) from the onset of storage modulus (G^I^). Excellent hot–wet performance of the resin system is favorable for aerospace applications. Figure 1 shows the chemical structures of the used reactive molecules.

During the prepreg production, the high-tenacity unidirectional carbon fiber rovings, HTS40 F13 12K (Toho Tenax Europa GmbH, Wuppertal, Germany) with 800-tex were used. As indicated before, each fiber roving consists of 12,000 single filaments. 

Neat MWCNTs (CNT-n) and plasma-treated MWCNTs (CNT-p) are provided by Future Carbon GmbH (Bayreuth, Germany) as extruded epoxy–MWCNT masterbatches without the hardener. For plasma treatment, a low-pressure rotary plasma reactor was used. The reactor rotates under vacuum, which whirls up the nanotube powder continuously. The plasma reacts with the CNT surface, and mainly hydroxyl and carboxyl groups are formed. The advantages of this method over conventional methods, such as acid functionalization or thermal treatment, are the dry process control, an extensive control of functionalization degree, and high throughput. Up to 2 kg of carbon nanotubes can be treated via plasma as one batch.

### 2.2. Production of Nanocomposites

**Figure 2:**
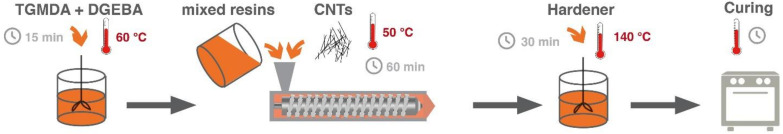
Production steps of neat and (multi-walled carbon nanotube) MWCNT-modified cured nanocomposites.

To produce a neat resin plate, 60 phr TGMDA was mixed with 40 phr of DGEBA (only in the resin part) via a laboratory scale mechanical stirrer at 60 °C and 500 rpm for 15 min, as shown in Figure 2. After this, the temperature of the resin was increased up to 140 °C and a stoichiometric amount of 4,4′-DDS hardener was added to the mixture which was then further mixed for 30 min to fully dissolve the hardener. The final mixture was cooled down to 80 °C under stirring in a water bath, and then degassed at 10 mbar for at least 10 min prior to curing. Finally, the degassed mixture was poured into a release agent treated steel mold and cured in a convection oven first at 180 °C for 160 min and post-cured at 200 °C for 60 min.

To produce MWCNT-modified epoxy nanocomposites, extruded MWCNT-TGMDA-DGEBA masterbatches delivered by Future Carbon GmbH were mixed at 140 °C with the stoichiometric amount of 4,4′-DDS for 30 min. Final mixtures were then cooled down, degassed, and cured as described before.

### 2.3. Production of Prepregs and Laminates

**Figure 3:**
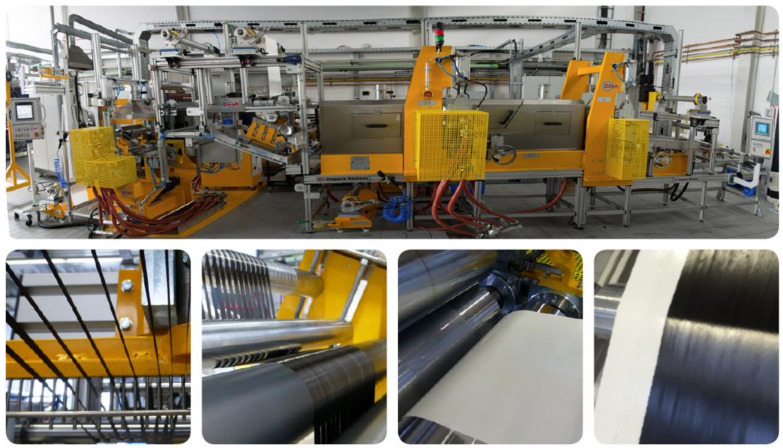
Above: Prepreg machinery at the Department of Polymer Engineering. Below, from left to right: sorting of single UD rovings, fiber pre-spreading unit, resin coating unit, and final produced prepreg, respectively.

The unidirectional (UD) prepregs were produced via hot melt processing at the laboratory scale prepreg impregnation machinery of the University of Bayreuth, shown in Figure 3.

At first, the unidirectional carbon fiber rovings were sorted and pre-spread. The resin film was coated at 70 °C on the siliconized carrier paper at the coating unit of the machinery. Finally, resin film and pre-spread fibers were impregnated to a final prepreg material via a heated calendar (5 bars, 100 °C). Usage of the 20 carbon fiber rovings during the prepreg production resulted in a unidirectional prepreg with approximately 200 mm width having an excellent fiber spreading and prepreg homogeneity as shown in Figure 3, right, which is a representative neat UD prepreg. 

In total, 26 prepreg layers with 140 to 150 gr/m^2^ areal weight were hand-laid up to achieve 3 mm thick laminates. Only the 2nd and the 25th layer were laid up as 90° to optimize the handling of the unidirectional structure. In the middle of the laminate in between 13th and 14th layer, a Teflon film with 50 mm width was inserted to initiate the crack propagation during interlaminar fracture toughness testing. 

The prepregs were cured and consolidated under vacuum and 7 bars external air pressure in a self-built autoclave. The temperature profile during curing is similar to the curing of the nanocomposites. It is important to mention that vacuum was applied until the gelation point of the resin, not the external pressure, to prevent extensive resin flow during curing.

### 2.4. Characterization Methods

#### 2.4.1. X-Ray Photoelectron Spectroscopy (XPS)

XPS spectra were obtained using a monochromatic Al-Kα X-ray source (ESCALAB 250Xi from Thermo Fisher Scientific, MA, USA) at Technical University Bergakademie Freiberg. A minimum of 2 samples of MWCNTs were analyzed before and after treatment.

#### 2.4.2. Interlaminar Shear Strength (ILSS) Testing 

ILSS of the prepreg laminates was measured according to DIN EN 2563 with a universal testing machine, Zwick Z1475 (Zwick GmbH & Co. KG, Ulm, Germany), at 23 °C and 55% relative humidity. A minimum of 10 samples per laminate were tested. 

#### 2.4.3. Fracture Toughness Testing of Cured Nanocomposites and Prepreg Laminates

The critical stress intensity factor (*K_Ic_*) was determined according to ISO 13,586 using compact tension (CT) specimens. The specimen length was 1.25w = 41.25 mm and the thickness d = 4 mm. For each specimen, a sharp crack was produced by tapping a razor blade into the machined V-notch. The tests were carried out using a universal testing machine, Zwick BZ2.5/TN1S (Zwick GmbH & Co. KG, Ulm, Germany). Crack opening displacement was measured with a clip extensometer (632.29F-30, MTS, Augsburg, Germany). 

Critical stress intensity factor (*K_Ic_*) and critical strain energy release rate (*G_Ic_*) of neat epoxy and nanocomposites were calculated according to Equations (1) and (2), respectively. *F_max_* is the maximum force required to propagate the crack, *d* is the sample thickness, *w* is the specimen length from the loading point, *f (a/w)* is a geometrical factor defined in ISO 13586, and finally *E_SH_* is the elastic modulus calculated according to the theory of Saxena and Hudak from the compliance during testing.

(1) $$K_{Ic}=\frac{F_{max}}{d\sqrt{w}}f\left(\frac{a}{w}\right)$$

(2) $$G_{Ic}=\frac{K_{Ic}^{2}}{E_{SH}}$$

The interlaminar fracture toughness of the cured prepreg laminates under mode I and mode II loading was then tested according to the DIN EN 6033 and 6034, respectively, using the same universal testing machine (Zwick BZ2.5/TN1S). The samples had a rectangular geometry of 250 × 25 × 3 mm^3^. In total, 2 N pre-load was applied to the samples, which were then tested with the testing speed of 10 mm/min. To eliminate any possible sample geometry and size dominating effects on the test results, the sample quality and thickness were kept similar for tested laminates.

#### 2.4.4. Scanning and Transmission Electron Microscopy 

The dispersion of MWCNTs in the cured epoxy resin was characterized using a LEO 922 A EFTEM transmission electron microscope (Carl Zeiss AG, Oberkochen, Germany) applying an acceleration voltage of 200 kV. Thin sections of 50 nm were cut on a Leica Ultracut microtome (Leica Biosystems GmbH, Nussloch, Germany) equipped with a glass knife.

Fracture surfaces of nanocomposites and prepreg laminates were examined with a Zeiss 1530 (Carl Zeiss AG, Oberkochen, Germany) scanning electron microscope having a field emission cathode. An acceleration voltage of 1.5 kV was set.

## 3. Results and Discussions

### 3.1. XPS Studies of MWCNTs and Their Morphology in Epoxy

The dispersion of nanoparticles, especially carbon nanotubes in a polymer matrix, is very challenging since an enormous dispersing energy input is necessary to overcome Van der Waals forces in between nanoparticles resulting agglomeration [15]. An increased compatibility of the MWCNTs with the epoxy matrix is especially expected to result in a qualitatively better distribution and dispersion of nanotubes in epoxy. 

X-ray photoelectron spectroscopy (XPS) was used to analyze the atomic concentration of carbon and oxygen at the very outermost surface of nanotubes before and after plasma modification.

According to the XPS results, the neat MWCNTs (CNT-n) consisted of 98.75% carbon atoms with 1.25% oxygen at the nanotube surface, as shown in Table 1. In addition, slightly above 80% of the bonding in between C and O was determined to be single, C–O. 

XPS studies of plasma-modified MWCNTs showed that the plasma treatment increased the oxygen content at the surface to 3.7%. In addition, the number of double bonds, C = O, increased from 17.6 to 30.3% after the treatment. 

**Figure 4:**
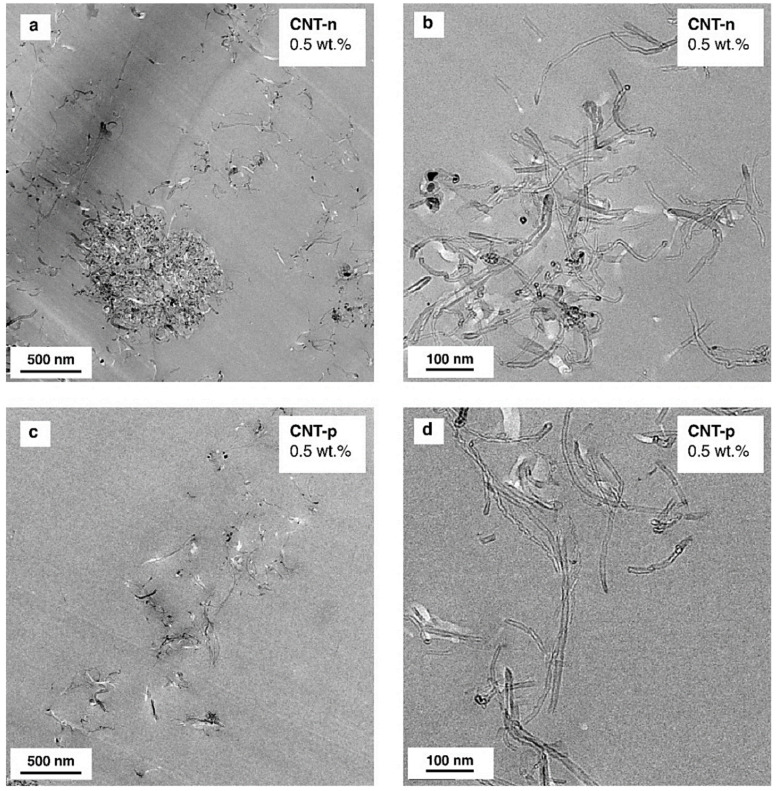
TEM micrographs of neat MWCNT (CNT-n) (**a**,**b**) and plasma-treated MWCNT (CNT-p) (**c**,**d**) nanocomposites. CNT content is 0.5 wt.%.

Transmission electron microscopy (TEM) was used to study the effect of the plasma treatment on the dispersion and distribution of MWCNTs in the cured systems. Figure 4 presents TEM micrographs of 0.5 wt.% CNT-n and CNT-p modified epoxy nanocomposites.

Although the extrusion process was chosen due to a very high input of dispersing energy, small agglomerates were inevitable for both types of MWCNTs. CNT-n modified epoxy nanocomposites contained agglomerates with a diameter of up to 750 nm, as shown in Figure 4a, as well as nanotubes as primary nanoparticles (Figure 4b). 

In the case of CNT-p modified nanocomposites, most of the plasma-treated nanotubes were dispersed and distributed in the resin as primary nanoparticles (Figure 4c,d). These results agree well with the literature, where the increased surface compatibility of the carbon nanotubes led to an improved nanotube dispersion and distribution in the two functional epoxy resin [21]. 

### 3.2. Fracture Toughness of Nanocomposites

**Figure 5:**
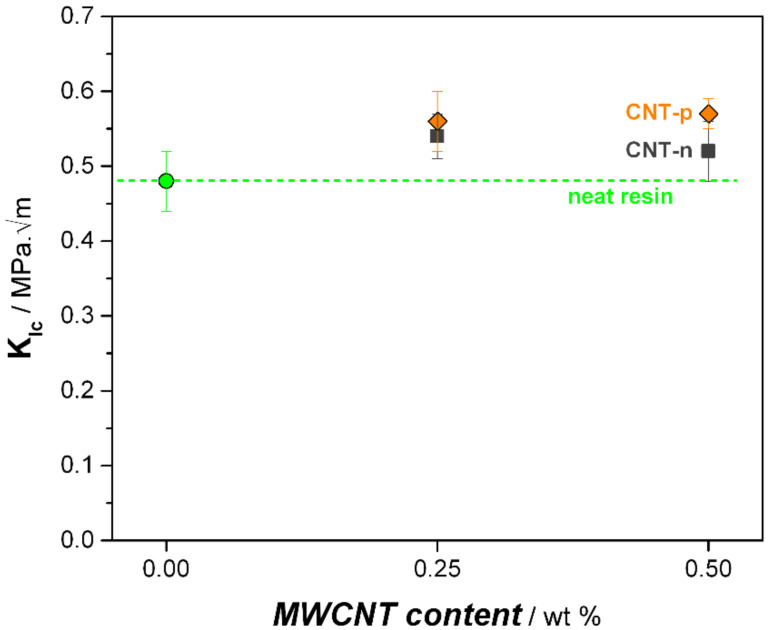
Fracture toughness of neat and MWCNT-modified nanocomposites.

The neat epoxy resin showed a brittle behavior which was reflected in the low *K_Ic_* value of 0.48 MPa·m^0.5^, shown in Figure 5. Both types of MWCNTs increased the toughness of the epoxy resin. The addition of the 0.25 wt.% CNT-n enhanced the toughness of the neat resin by 10%. The higher addition of CNT-n did not improve the toughness of the resin any further.

The influence of CNT-p on the toughness of the epoxy resin was more pronounced compared to the neat nanotubes. In total, 0.25 wt.% CNT-p modified epoxy resin showed a *K_Ic_* value of 0.56 MPa.m^0.5^. In contrast to the neat MWCNTs, further addition of CNT-p (0.5 wt.%) resulted in a 20% improved toughness compared to the neat epoxy resin. 

**Figure 6:**
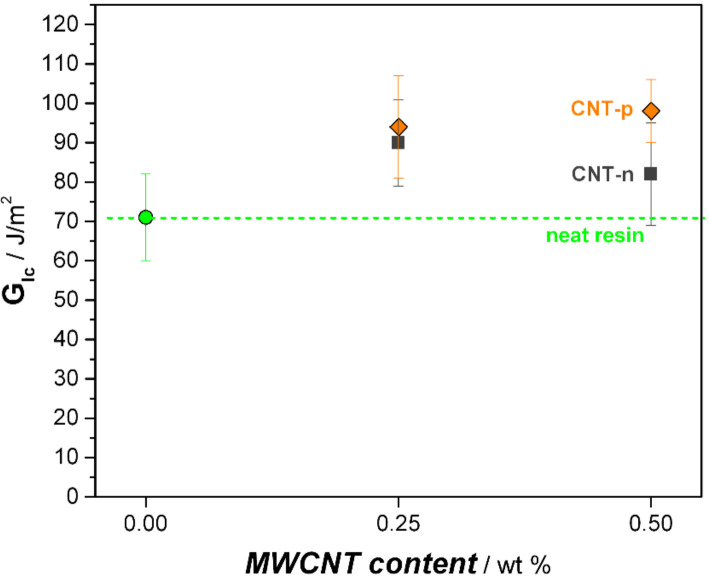
Strain energy release rates of neat and MWCNT-modified nanocomposites.

Figure 6 shows the strain energy release rates (*G_Ic_*) of epoxy nanocomposites. The neat resin shows a relatively low energy release rate (*G_Ic_*) of 71 J/m^2^. Similarly to the impact of MWCNTs on the critical stress intensity factor of epoxy resin, the addition of both CNTs, regardless of the surface modification, enhanced the strain energy release rate. The addition of only 0.25 wt.% CNT-n increased the strain energy release rate 27% (90 J/m^2^) compared to neat system, whereas the further addition of CNT-n resulted in a slight decrement of *G_Ic_* with the value of 82 J/m^2^. As stated by Ganguli et al. [12], randomly distributed and oriented neat MWCNTs show the potential to enhance the fracture toughness of highly cross-linked epoxy resin by increasing the fracture surface area and the absorbed energy.

Plasma-treated nanotubes led to a steady enhancement of strain energy release rate with increasing filler content, which was up to 38% higher *G_Ic_* at 0.5 wt.% CNT-p content. These observations pointed out the higher impact of surface-modified carbon nanotubes on the toughness of epoxy resins. The results agree well with previous literature. As stated by Gojny et al. [17], enhanced surface compatibility of CNTs with the host polymer favors the toughening effect of nanotubes further.

### 3.3. Interlaminar Shear Strength of Prepreg Laminates

**Figure 7:**
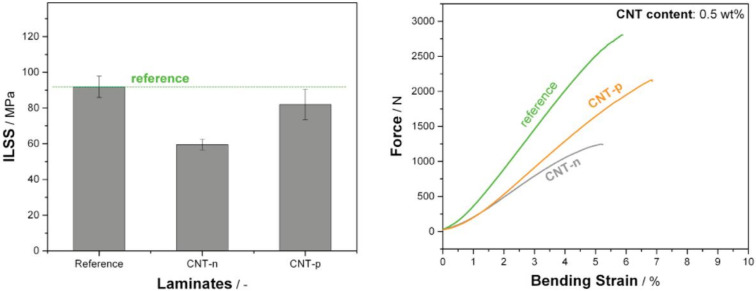
(**a**) Interlaminar shear strength (ILSS) of laminates. (**b**) F–ε (force–strain) diagrams. MWCNT contents are 0.5 wt.%.

Results of short beam three-point bending testing of prepreg laminates are shown in Figure 7.

Neat prepreg laminate shows 91 MPa interlaminar shear strength, whereas the addition of both types of nanotubes resulted in a deterioration of ILSS compared to the neat system. The addition of 0.5 wt.% neat nanotubes (CNT-n) resulted in a 33% lower ILSS compared to reference. However, the inclusion of CNT-p lowered the ILSS of the neat polymer only slightly. Godara et al. [23] reported as well that the addition of various types of CNTs was deteriorative for interlaminar shear strength of prepreg laminates. It was noted that CNTs act as failure initiation zones under this complex loading condition. 

The lower matrix compatibility of CNT-n and pronounced agglomeration is therefore claimed to be responsible for the lower ILSS. 

### 3.4. Interlaminar Fracture Toughness of Prepreg Laminates

Table 2 presents the strain energy release rates for MWCNT-modified epoxy nanocomposites and prepreg laminates.

Compared to neat resin, the neat UD epoxy–carbon fiber laminate shows a threefold higher *G_Ic_* value, which is attributed to the bridging of the fibers. Unlike its epoxy nanocomposite, CNT-n addition in the prepreg laminate resulted in 13% lower interlaminar fracture toughness compared to reference laminate. On the other hand, 0.5 wt.% CNT-p modified laminate showed approximately 40% higher fracture toughness. 

**Figure 8:**
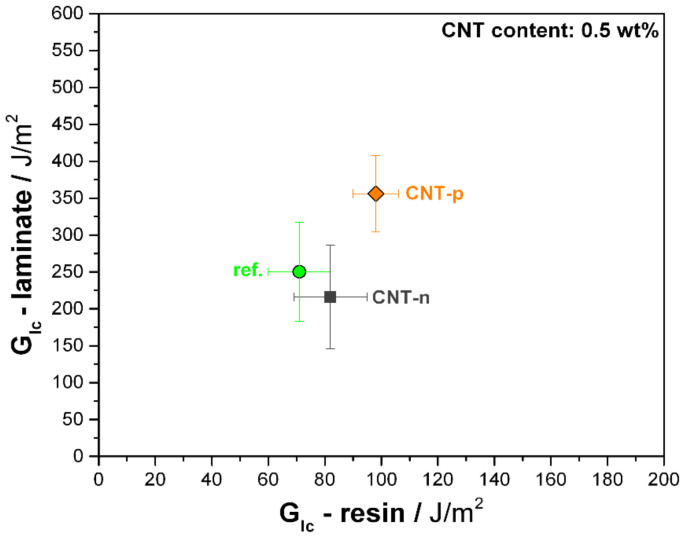
*G_Ic_* of neat and 0.5 wt.% MWCNT-modified epoxy resin and UD prepreg laminates.

As already reported in literature, the increased fracture toughness of resin does not necessarily promise an improvement of the delamination toughness of its composite, especially to a comparable degree [24]. Correlations in Figure 8 reveal the detrimental effect of neat MWCNTs (CNT-n) on the delamination toughness of its epoxy–carbon fiber prepreg laminate, whereas plasma-modified nanotubes resulted in a rather comparable toughening effect in resin and its prepreg laminate.

**Figure 9:**
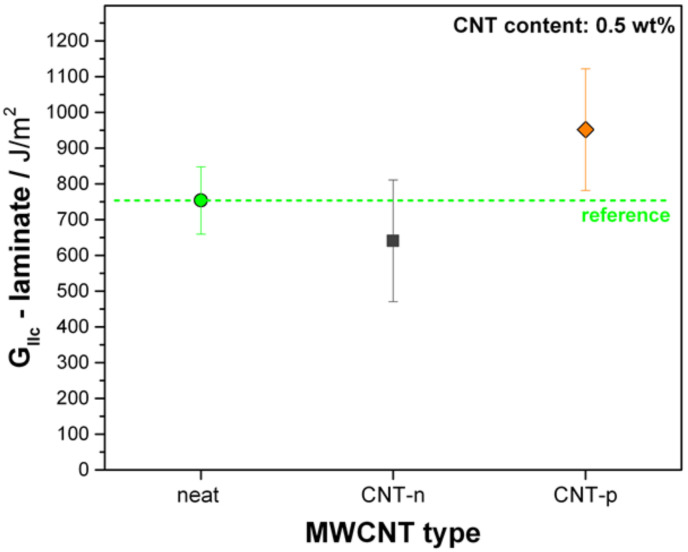
G_IIc_ of neat and 0.5 wt.% MWCNT-modified UD prepreg laminates.

The same trend is observed under quasi-static mode II loading, as shown in Figure 9. Unidirectional carbon fiber-neat epoxy prepreg laminate has 753 ± 90 J/m^2^ mode II critical strain energy release rate. The 0.5 wt.% CNT-n modified prepreg laminate showed a 15% lower G_IIc_ compared to the reference laminate. The same content of plasma-modified CNTs led to an 26% improvement of the mode II toughness with measured G_IIc_ of 950 ± 170 J/m^2^.

Consequently, plasma modification directly influenced the impact of carbon nanotubes on the toughness of epoxy resin and its UD carbon fiber prepreg laminates. Neat MWCNTs lowered the interlaminar shear strength and interlaminar fracture toughness tremendously, whereas plasma-modified nanotubes enhanced the toughness with a slight decrease of the interlaminar shear strength, pointing out the importance of the nanotube-matrix compatibility on mechanical properties.

### 3.5. Micromechanical Toughening Mechanisms in Prepreg Laminates

#### 3.5.1. Mode I Loading

**Figure 10:**
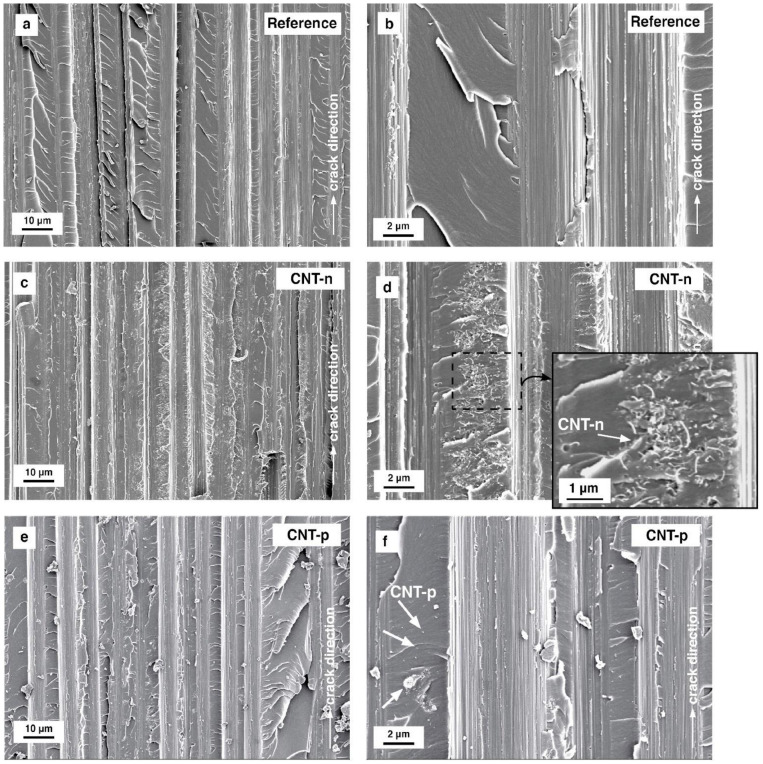
Scanning electron micrographs of fracture surfaces from (**a**) and (**b**) neat, (**c**) and (**d**) 0.5 wt.% CNT-n modified, and (**e**) and (**f**) 0.5 wt.% CNT-p modified epoxy–carbon fiber UD prepreg laminates tested under mode I loading. White arrows indicate the crack propagation direction.

As shown in Figure 10, the fracture surface micrographs of the reference prepreg laminate have a relatively smooth topography with a high number of microcracks in the resin-rich zones. Although excellent fiber–resin adhesion is observed (see Figure 10b), carbon fiber pull-out was seen at the fracture surface of the neat prepreg laminate (Figure 10a).

In the case of CNT-n modified prepreg laminates, a strong agglomeration of CNT-n in resin-rich areas can be seen, which can be observed in Figure 10d. The microcracking was not suppressed by these agglomerates. In addition, similar to the reference laminate, a high number of carbon fiber pull-outs was observed at the fracture surface. Surfaces of pull-out regions were slightly covered with CNTs. By this means, the extensive agglomeration of CNT-n is suspected to lower the carbon fiber–matrix adhesion which reflects itself in the deteriorated interlaminar fracture resistance. 

Although agglomerates up to 1 μm were still observable in the resin-rich regions, CNT-p was dispersed mostly as single particles. Parallel to microcracking of resin and fiber pull-out, the pull-out of single CNT-p nanotubes was observed in resin-rich areas, shown in Figure 10f. 

#### 3.5.2. Mode II Loading

**Figure 11:**
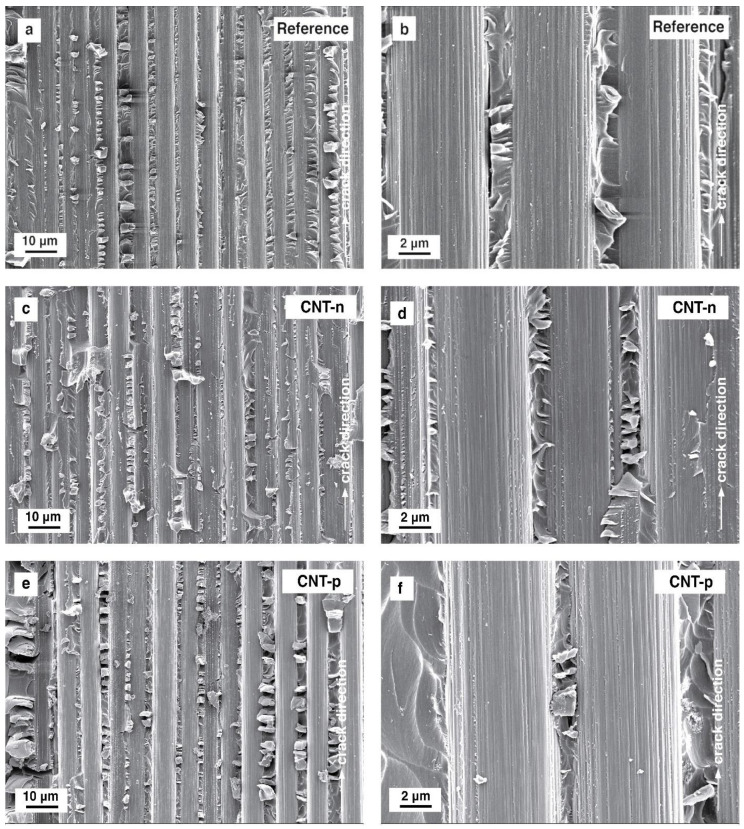
Scanning electron micrographs of mode II fracture surfaces from (**a**) and (**b**) neat, (**c**) and (**d**) 0.5 wt.% CNT-n modified, and (**e**) and (**f**) 0.5 wt.% CNT-p modified epoxy–carbon fiber UD laminates.

Figure 11 presents the micrographs from fracture surfaces of the prepreg laminates tested under mode II loading.

The fracture surfaces of all tested laminates, shown in Figure 11a, c and e reveal the carbon–fiber pull-out. A very bare fiber surface can be observed for all prepreg laminates, although under mode I loading, resin fragments were observed at the fiber surfaces.

Under mode II loading of fiber-reinforced composites, hackles are formed by the microcracking, followed by microcrack collapse, and final hackle formation due to the shear stresses at the resin-rich zones [25]. The neat epoxy prepreg system shows clearly observable hackles of the deformed matrix polymer, which can be seen in Figure 11a,b. In the case of 0.5 wt.% CNT-n modified prepreg laminate, repetitive hackle formation is slightly suppressed, as shown in Figure 11c. In addition, the altitude of the hackles is qualitatively lower compared to the reference laminate. At the fracture surfaces of CNT-p modified prepreg laminate, hackle formation was not suppressed but observed to be more pronounced. 

Recently, Assami et al. [26] investigated the effect of the neat carbon nanotubes and their morphology, especially the effect of their orientation in epoxy on the stiffness and delamination resistance of epoxy–fiber-reinforced composites by a multiscale modeling approach based on the finite element model (FEM). FEM analysis suggested that even a low CNT content (1 wt.%) in inter-ply regions of a unidirectional composite results in an enhanced stiffness and delamination resistance, under in-plane shear loading. Although it is at the moment industrially not feasible, 45° orientation of the CNTs compared to the loading axis is suggested to be favorable for increased delamination resistance under shear loading. On the other hand, the detrimental effect of the neat MWCNTs observed in this work is attributed to the extensive agglomeration. Therefore, the importance of the surface modification of MWCNTs is shown here to be crucial for composite applications. 

## 4. Conclusions

Carbon nanotubes with and without plasma treatment were added to a highly cross-linked epoxy resin system and its unidirectional carbon fiber prepreg laminates. Plasma treatment led to an increase of oxygen atom concentration at the surface of MWCNTs. Although the agglomeration was inevitable for both types of CNTs, TEM micrographs revealed an improved dispersion of MWCNTs in the resin after plasma modification. 

The neat epoxy resin showed a very brittle behavior. The addition of a low number of MWCNTs resulted in an improvement of quasi-static fracture toughness of the resin. Furthermore, the plasma treatment was beneficial to promoting higher energy dissipation during crack propagation due to an increased number of CNT pull-out. 

During prepreg production, fully impregnated unidirectional carbon fiber prepregs of neat and MWCNT-modified resins were produced. Extensive agglomeration of neat MWCNTs (CNT-n) seems to have a detrimental effect on the interlaminar fracture toughness under mode I and mode II loading by possibly deteriorating the matrix–fiber adhesion and suppressing hackle formation, respectively. Plasma modification prohibited this excessive CNT agglomeration. Therefore, no detrimental effect of plasma-modified CNTs was observed regarding the interlaminar fracture toughness of laminates. These findings emphasize the importance of an improved compatibility and dispersion of CNTs in interlaminar regions of unidirectional laminates to fully benefit from the toughening mechanisms of nanotubes.

## Figures and Tables

**Table 1:** X-ray photoelectron spectroscopy of neat and plasma-treated MWCNTs.

C, O and Their Bonding	Neat MWCNTs	Plasma-Treated CNTs
Carbon (%)	98.75 ± 0.45	96.30 ± 0.28
Oxygen (%)	1.25 ± 0.45	3.70 ± 0.28
C–O bonds (%)	82.35 ± 3.45	69.65 ± 2.05
C=O bonds (%)	17.65 ± 3.45	30.35 ± 2.05

**Table 2:** Strain energy release rates of MWCNT-modified epoxy nanocomposites and prepreg laminates. CNT content is 0.5 wt.% for both types of CNTs.

Resin -	*G_Ic_*–Resin J/m^2^	*G_Ic_*–Laminate J/m^2^	*G_IIc_*–Laminate J/m^2^
Reference	71 ± 11	250 ± 67	754 ± 94
CNT-n modified	82 ± 13	216 ± 70	641 ± 170
CNT-p modified	98 ± 8	356 ± 52	952 ± 170

## Data Availability

The data presented in this study are available on request from the corresponding author.
